# A Medico-Legal Treatise on Malpractice and Medical Evidence

**Published:** 1871-05

**Authors:** 


### Book Notices.

A Medico-Legal Treatise on Malpractice and Medical Evidence, comprising the elements of Medical Jurisprudence. By John J. Elwell, M.D. Third edition, revised and enlarged. Baker, Voorhis & Co., publishers, 66 Nassau Street, New York.

The former editions of this work received the highest commendations from the leading medical and legal authorities of this country and England.

The opening chapter, devoted to the subject of malpractice, is of especial interest and value. The subjects of Amputations, Fractures, and Dislocations, out of the treatment of which grow the great majority of suits for malpractice, are taken up, and an exhibit of the present state of the science in regard to the surgery of these cases presented; that just what should be rightfully expected and required of the surgeon, may appear as far as possible, and what should excuse an imperfect result in his treatment. A digest of Prof. Frank H. Hamilton's able and valuable report on "Deformities after Fractures," and a chapter on the responsibilities of druggists, with the leading cases where they have been sued, close the part of the work devoted to civil malpractice.

Criminal malpractice, including the subject of abortion, is next presented, together with the leading adjudicated cases.

Part Second is devoted to the consideration of the leading points and subjects in medical evidence.

Infanticide, wounds, rape, and coroner's inquests, close up the subjects treated in this work.

